# Cognitive maps in the wild: revealing the use of metric information in black howler monkey route navigation

**DOI:** 10.1242/jeb.242430

**Published:** 2021-08-13

**Authors:** Miguel de Guinea, Alejandro Estrada, K. Anne-Isola Nekaris, Sarie Van Belle

**Affiliations:** 1 School of Social Sciences, Oxford Brookes University, Oxford, OX3 0BP, UK; 2 Movement Ecology Lab, Department of Ecology, Evolution and Behavior, The Hebrew University of Jerusalem, Jerusalem 91904, Israel; 3 Institute of Biology, National Autonomous University of Mexico, CP 04510 Mexico City, Mexico; 4 Department of Anthropology, University of Texas at Austin, Austin, TX 78712, USA

**Keywords:** *Alouatta pigra*, Labelled graph map, Route network, Network analysis, Revisiting behaviour, Spatial cognition

## Abstract

When navigating, wild animals rely on internal representations of the external world – called ‘cognitive maps’ – to take movement decisions. Generally, flexible navigation is hypothesized to be supported by sophisticated spatial skills (i.e. Euclidean cognitive maps); however, constrained movements along habitual routes are the most commonly reported navigation strategy. Even though incorporating metric information (i.e. distances and angles between locations) in route-based cognitive maps would likely enhance an animal's navigation efficiency, there has been no evidence of this strategy reported for non-human animals to date. Here, we examined the properties of the cognitive map used by a wild population of primates by testing a series of cognitive hypotheses against spatially explicit movement simulations. We collected 3104 h of ranging and behavioural data on five groups of black howler monkeys (*Alouatta pigra*) at Palenque National Park, Mexico, from September 2016 through August 2017. We simulated correlated random walks mimicking the ranging behaviour of the study subjects and tested for differences between observed and simulated movement patterns. Our results indicated that black howler monkeys engaged in constrained movement patterns characterized by a high path recursion tendency, which limited their capacity to travel in straight lines and approach feeding trees from multiple directions. In addition, we found that the structure of observed route networks was more complex and efficient than simulated route networks, suggesting that black howler monkeys incorporate metric information into their cognitive map. Our findings not only expand the use of metric information during route navigation to non-human animals, but also highlight the importance of considering efficient route-based navigation as a cognitively demanding mechanism.

## Abstract (precis)

**Highlighted Article:** Black howler monkeys rely on route-based cognitive maps, which constrain their movement decisions, but likely incorporate metric information to navigate more efficiently along frequently used routes.

## INTRODUCTION

Living in tropical forests involves coping with a complex matrix of environmental information characterized by highly variable spatial and temporal distributions of food resources (Janmaat et al., 2016; Levey, 1988; Morales et al., 2010). Animals have developed a wide variety of navigational strategies to make use of environmental information and reach relevant biological locations (e.g. celestial cues to orientate migratory routes: Foster et al., 2018; visual landmarks associated to foraging sites: Zeil, 2012; path integration using nest location as reference: Heinze et al., 2018). Among these navigational strategies, some species generate internal representations of the space wherein they live, known as ‘cognitive maps’ (Tolman, 1948). The level of sophistication of these cognitive maps has been argued to be analogous with the cognitive capacity of a species (Poucet, 1993; Tolman, 1948; Warren, 2019). In turn, sophisticated cognitive maps likely support flexible and efficient movement patterns that enhance foraging efforts of individuals or groups (Behrens et al., 2018; Milton, 1981).

Even though the concept of cognitive maps has been a subject of debate during the last decades (Bennett, 1996; Collett and Collett, 2006; Warren, 2019), there has been a consensus postulating that navigation flexibility can be considered along a continuum with one end representing Euclidean maps and the other route-based maps (Byrne, 2000; Warren, 2019; Warren et al., 2017). Euclidean maps are built upon the knowledge of distances and directions among locations within animals' habitats in a globally consistent coordinate system (Gallistel, 1990; O'Keefe and Nadel, 1978). Calculating novel paths or detours between known locations is supported by Euclidean maps, providing a high level of navigation flexibility (McNaughton et al., 2006; but see Warren, 2019). Alternatively, route-based maps are composed of a series of habitually used routes that interconnect pairs of relevant locations – called ‘nodes’ – across an animal's home ranges (i.e. route networks; Perna and Latty, 2014; Trapanese et al., 2019). Nodes are often associated with emergent trees or ridges, where animals likely increase their visual access to the surroundings and may decide where to go next (Presotto et al., 2018). Even though route-based maps constrain an animal's movement options to a series of pre-established routes (Di Fiore and Suarez, 2007), they have been widely reported across animal taxa (birds: Guilford and Biro, 2014; mammals: Newmark and Rickart, 2012; Trapanese et al., 2019; insects: Mangan and Webb, 2012; Baddeley et al., 2012). Chrastil and Warren (2014) indicated that humans rely strongly on route-based maps but incorporate metric and angular information into their movement decisions. After having travelled repeatedly to specific targets, individuals benefited from establishing a set of highly efficient habitual routes that would support travelling along the shortest path to reach multiple targets from multiple starting locations (Warren, 2019). Thus, route-based maps could differ in their structure depending on an individual's ability to incorporate metric information into movement decisions (Chrastil and Warren, 2014; Warren, 2019). Route-based maps lacking metric information were categorized as ‘topological maps’ while those incorporating metric information were categorized as ‘labelled graph maps’, in which the latter would provide a certain level of navigational flexibility not only to humans but also to non-human animals (Presotto et al., 2018; Ericson and Warren, 2020). Therefore, in order to disentangle the navigational strategy developed by wild animal populations and avoid misinterpretation of results, it is necessary to explore in detail multiple properties of their movement patterns (Cheung et al., 2014; Bertolani, 2013; Janmaat, 2019).

To infer the cognitive map adopted by wild animals, researchers typically extract a combination of metrics from their ranging patterns (Di Fiore and Suarez, 2007; Harten et al., 2020; Noser and Byrne, 2007; Porter and Garber, 2013; Presotto and Izar, 2010; Presotto et al., 2019). First, if an animal navigates using a route-based map, travel paths are expected to overlap each other describing a series of habitual routes (Di Fiore and Suarez, 2007; Presotto et al., 2019). Increasing frequency in the overlap of travel paths can be the result of random movement in which the moving agent repeats a series of heuristics when navigating (Benhamou, 2007). To determine whether the observed ranging patterns correspond to spatial knowledge instead of heuristic rules, researchers examine metrics at both small and large spatial scales (i.e. inside and outside the visual detection distance of food resources; Porter and Garber, 2013). At a small scale, examining the number of arrival and departure directions to revisited biologically meaningful locations might indicate whether the study subject navigates using a route-based map (i.e. if arrival angles are clustered in one or few directions around these revisited sites) or a Euclidean map (i.e. if arrival angles are not clustered around revisited sites; Toledo et al., 2020). Similarly, at a large scale, route-based maps would lead to highly deviating travel trajectories towards out-of-sight targets owing to the shape of the habitual routes (Porter and Garber, 2013). Subjects relying on Euclidean maps, however, can increase the linearity of their travel trajectories to gain access to food resources before other group members when travelling in large groups (i.e. increased intragroup competition; Tujague and Janson, 2017; Salmi et al., 2020). For instance, Egyptian fruit bats (*Rousettus aegyptiacus*) travel in a highly linear manner towards preferred fruit trees regardless of origin, goal type or distance from the destination (Toledo et al., 2020), even during the first months of their lives (Harten et al., 2020). Therefore, the combination of these metrics – path overlap, number of path directions and linearity – at different spatial scales has the potential of highlighting the consistent use of a specific cognitive map by a species.

Additionally, if the evidence suggests that the study subjects use a route-based map, the next step is to identify whether their cognitive map is a topological map or a labelled graph map (Warren et al., 2017; Ericson and Warren, 2020). Determining the use of metric information during navigation in humans can be done through interviews (Byrne, 1979; Foo et al., 2005; Ericson and Warren, 2020). In contrast, non-human animal studies require examining the structure of route-based maps to infer the use of metric information during navigation (Perna and Latty, 2014; Presotto et al., 2018). By examining the complexity in the number of connections among pairs of nodes within a network (i.e. habitual route segments), we can infer and compare the sophistication of the cognitive process required to generate and navigate in different networks (Gallotti et al., 2016; Janmaat et al., 2021). First, the strength of a route network indicates the involvement of each individual node in the overall activity of the network (Barthélemy, 2011; Soh et al., 2010). Animals navigating through route networks with a high level of strength have more options to combine route segments to reach specific locations, which likely minimizes the time and energy spent travelling. Second, calculating the mean shortest path length to travel from each node to any other node within the network indicates the navigation efficiency of a network (Barthélemy, 2011). Route networks with low mean shortest path lengths were likely optimized over time by incorporating metric information and consequently selecting the shortest paths across relevant locations in an area (Gastner and Newman, 2006). Third, the centrality of a network indicates whether an individual is required to always travel through a fixed number of nodes to reach their goals regardless of their starting location (Borgatti, 2005). For non-central place foragers, route networks with low centrality would support more flexible movement patterns than highly centralized networks, which might lead to increased time and energy spent travelling (Barthélemy, 2011). Overall, we would expect topological maps to show low levels of strength, long shortest path lengths and high centrality, while we would expect labelled graph maps to show high strength, short shortest path lengths and low centrality.

The black howler monkey (*Alouatta pigra* Lawrence 1933) is a quadrupedal, arboreal primate that travels in highly cohesive social units across the middle and upper strata of the canopy (Van Belle et al., 2013; Youlatos et al., 2015). The physiological adaptations of howler monkeys support highly flexible dietary patterns, which in addition to feeding on leaves, flowers and stems, are characterized by fruit consumption that ranges between ca. 10% and ca. 80% of their feeding time (Dias and Rangel-Negrín, 2015). They experience long retention rates in their digestive system, which reduces the availability of metabolic energy (Amato and Righini, 2015; Milton, 1981). Hence, developing a navigation strategy that would minimize energy expenditure while travelling would be highly beneficial for the species (de Guinea et al., 2019). While the evolution of sensorial adaptation such as systematic trichromatic colour vision benefits the acquisition of food resources (Jacobs et al., 1996), howler monkeys have been shown to rely on their cognitive skills during foraging as well (Fortes et al., 2015). For instance, Hopkins (2016) demonstrated that mantled howler monkeys (*Alouatta palliata*) in Panama incorporated information regarding the spatiotemporal distribution of fruit availability in their movement decisions. Similarly, different species of howler monkey have been shown to incorporate memories associated with social (Hopkins, 2013; Van Belle and Estrada, 2020), ecological (Plante et al., 2014; Hopkins, 2016) and physical (Ceccarelli et al., 2019) information from their surroundings in order to enhance their spatial performance under varying environmental conditions (de Guinea et al., 2021a).

**Fig. 1.**
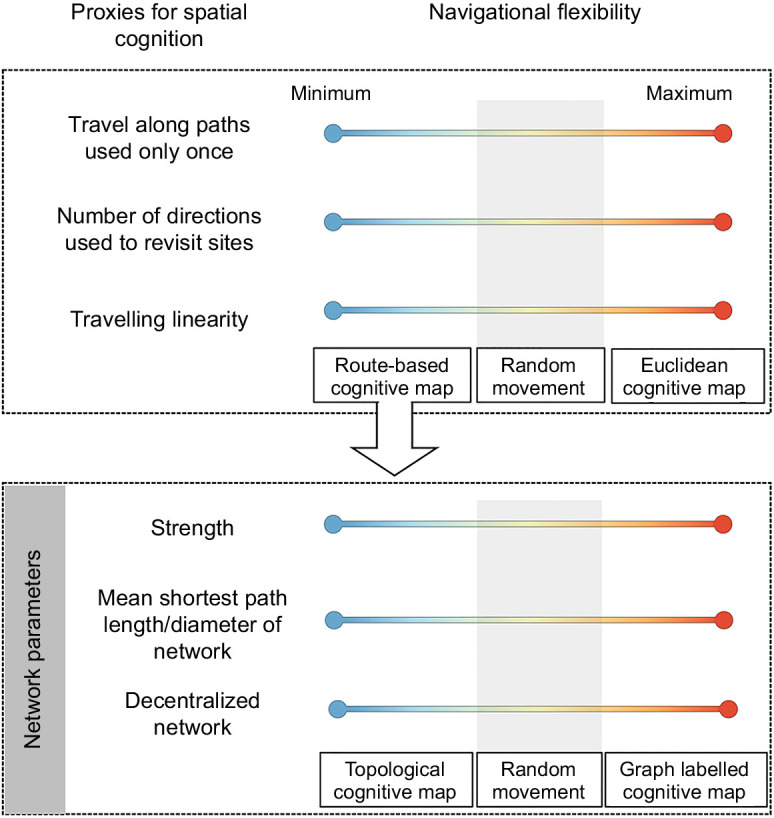
Summary of the cognitive hypotheses tested comparing black howler monkeys and simulated random-walk movement patterns.

Here, we made use of a novel approach to examine the navigation flexibility of black howler monkeys by testing a series of cognitive hypotheses in their movement patterns using simulated random-walk movement patterns as controls. Previously, computationally generated patterns of random movement have been used to reveal cognitive aspects of migratory routes (Bracis and Mueller, 2017) and home range space use patterns in wild populations (Gautestad et al., 2013). We examine whether the movement patterns of black howler monkeys differ from the movement patterns of simulated agents in their tendency to: (1) travel along paths used only once; (2) reach and depart from revisited sites using few or multiple directions; and (3) increase the linearity of their travel trajectories under variant conditions of knowledge, intragroup competition and resource availability. In addition, we examined the structural properties (i.e. strength, mean shortest path length and centrality) of both observed and simulated route networks to determine whether black howler monkeys rely on a labelled graph map or a topological map to navigate (Fig. 1).

## MATERIALS AND METHODS

### Study site and groups

We conducted the study at Palenque National Park (17°27′52″–17°30′10″N, 92°01′48″–92°01′48″W), Mexico, which is predominantly composed of primary, tall, evergreen tropical rainforest (Diaz-Gallegos, 1996). The average annual temperature fluctuates around 26°C and annual rainfall is asymmetrically distributed between a rainy season (June–October, mean precipitation 1971–2000 *P*=294.7±71.26 mm) and a drier season (November–May, mean precipitation 1971–2000 *P*=134.6±56.95 mm; CONAGUA, http://smn.cna.gob.mx/, accessed from the historical records). We selected five neighbouring groups of black howler monkeys (i.e. Balam, Motiepa, Naha, Pakal and Unites), which were composed of 4 to 11 individuals and had home ranges varying between 7.1 and 15.3 ha (de Guinea et al., 2019).

Permission to conduct research in Palenque National Park was granted by SEMARNAT (Dirección General de Vida Silvestre de la Secretaria del Medio Ambiente y Recursos Naturales de México) under permit SEMARNAT SGPA/DGVS/05700/16.

### Data collection

We collected behavioural and ranging data from September 2016 through August 2017. For four consecutive days (ca. 05:00–17:00 h) a week, we collected data on two focal groups simultaneously. Thus, we collected 3104 h of behavioural data (*N=*59.8±2.17 days/group; *N*=620.71±29.586 h/group, means±s.e.m.). Throughout the day, we conducted scan samples at 15-min intervals to record the behaviour of all visible group members (e.g. loud calling, foraging, resting, socializing). In addition, whenever one or more group members fed in a tree for more than 5 min, we recorded the duration of the feeding bout, the item fed on and the plant species. We also marked these feeding trees, recorded their diameter at breast height and estimated their height (mean±s.e.m. *N=*356.2±66.37 feeding trees/group).

We used a GPS Garmin 64S (mean±s.e.m. GPS error: 6.6±2.3 m) to collect locations along black howler monkeys' travel paths every 20 m below the estimated centre of the group. We collected movement data from the moment two or more group members moved into a neighbouring tree or further until at least two members of the group engaged in an activity other than travelling for more than 5 min, which was defined as a travel bout (Van Belle et al., 2013). Because black howler monkeys travel, rest and forage in cohesive social units that do not separate ≥15 m (Van Belle and Estrada, 2020), we tracked each group as a unit instead of tracking all individuals within the same group (Van Belle et al., 2013). We recorded a total of 1528 travel bouts, with a mean±s.e.m. of 305.6±43.9 travel bouts per group (range: 250–368 travel bouts per group).

### Data analyses

We estimated the home range of each study group using a fixed kernel density estimation (KDE) method (Worton, 1989) with the package adehabitatHR 0.3.24 (Calenge, 2006) in R 6.0. We defined a group's home range as the 95% KDE isopleth and a core area as the 50% KDE isopleth (Van Belle et al., 2013). To compare the tendency of black howler monkeys to overlap travel paths with what would be expected if they travelled randomly within their home range, we firstly simulated correlated random-walk paths within the estimated home range isopleths – excluding areas that were not characterized by a continuous canopy coverage – using the packages SiMRiv 1.0.3 (Quaglietta and Porto, 2019) and sf 0.7–6 (Pebesma, 2019). Simulations within each study group’s home range used the group's maximum step lengths [i.e. the upper limit of the distribution of realized step length (straight line between consecutive ranging points) by study groups; Quaglietta and Porto, 2019; Balam: 12 m; Motiepa: 18 m; Naha: 37 m; Pakal: 12 m; Unites: 17 m] and the turning angle concentration parameter of the wrapped normal distribution between steps (Balam: 0.004 deg; Motiepa: −0.031 deg; Naha: 0.025 deg; Pakal: −0.025 deg; Unites: −0.048 deg; function sampleMovement from the package SiMRiv; Fig. S1). We chose the same mean number of steps travelled per day from each simulation (Balam: 58; Motiepa: 58; Naha: 64; Pakal: 61; Unites: 59) to approximate the mean observed daily path lengths for each study group (mean simulated path length: Balam: 731±109 m; Motiepa: 631±143 m; Naha: 1182±103 m; Pakal: 891±230 m; Unites: 855±117 m). We implemented our simulations by specifying that simulated agents would represent two behavioural states (i.e. ‘travelling’ as long steps and smooth turning angles; ‘foraging’ as small steps and marked turning angles) based on our experience in the field (Kareiva and Shigesada, 1983). Because we observed our study groups during four consecutive days a week, ranging behaviour was spatially autocorrelated within a weekly sampling unit (Cushman, 2010). Therefore, we equally aimed to generate a spatial autocorrelation in the simulated movement by running four consecutive days of simulations at a time (starting at a randomly selected location within the group's home range). These were repeated as many times as the number of weekly sampling units we observed for each group (Balam=10; Motiepa=15; Naha=17; Pakal=15; Unites=13).

Secondly, we generated a route network based on the ranging patterns of either the study groups or the simulated random-walk agents. For this, we overlaid all daily travel paths recorded during the same week per group onto a raster map of the area and checked their concordance (Di Fiore and Suarez, 2007). Whenever a daily path fell within a 10 m buffer of another daily path of that week for at least 15 m without deviating more than 45 deg from the other path, we considered it as the same travel segment (Presotto and Izar, 2010). We selected these parameters to be consistent with previous research and to control for GPS accuracy and travel directionality (Bebko, 2018; Porter et al., 2020). Food resources in rainforests can occur for a short period in the same location within the same week (Janmaat et al., 2016), which can lead to an overestimation of the frequency of reused routes (Presotto and Izar, 2010). Hence, we constructed for each sampled week ‘weekly paths’ by excluding the reuse of paths within the same week. Subsequently, each group's weekly paths were overlaid on top of each other, and we repeated the same procedure as described above to determine across how many weeks route segments were reused. We defined the habitual route network of each group as path segments used during at least two separate observation weeks. For the simulated movement, we followed the same protocol by describing path overlap within simulated weekly paths and, subsequently, determining overlap among all simulated weekly paths per group. To examine whether black howler monkeys reuse paths more or less than would be expected by chance, we compared the overall distance travelled within the observed route networks and the simulated ones. For this, we calculated and compared the distance travelled inside and outside of a 10 m buffer around all route segments used at least twice. In addition, we tested whether the frequencies with which habitual routes were used by black howler monkeys were higher than that of simulated agents. We weighted the frequencies with which route segments were used by their proportion, which we calculated as the length of the habitual route segment divided by the total length of the travel paths.

To test whether black howler monkeys approached and departed from feeding trees using a smaller number of routes (i.e. directions) than simulated agents did, we assumed a 20 deg threshold in between all the arrival and departure directions towards and from the same feeding tree (Urbani, 2009). We calculated the total number of routes used to reach feeding trees that were visited at least five times and divided it by the total number of visits to these feeding trees. Because simulated random-walk agents were not revisiting physical feeding trees, we used the R package recurse (Bracis et al., 2018) to identify highly revisited locations (Fig. S2). We selected a radius of 40 m as the simulated agent's visual range mimicking black howlers' estimated visual range in rainforests (Hopkins, 2011). Subsequently, we randomly selected an equal number of simulated locations that were revisited at least 5 times in each home range to the observed number of revisited feeding trees for each study group. We calculated the number of different directions used to reach these locations following the same protocol as described for the observed data. We compared both the accumulated number of directions and the travelling frequency each direction was used between the observed and the simulated datasets.

We calculated linearity by dividing the actual distance travelled during each travel bout by the straight line between the start and end locations (Jang et al., 2019; Normand and Boesch, 2009; Valero and Byrne, 2007). When calculating these distances, we included differences in elevation to account for our study site's topography (see Fig. S3). We selected travel bouts longer than the estimated visual range of black howler monkeys within a dense rainforest to minimize the influence of visual cues in their movement decisions (i.e. 40 m; Hopkins, 2011; Milton, 1981). In addition, we controlled for the influence of substrate availability to travel (i.e. inter-tree connectivity) by excluding travel bouts that intercepted a canopy gap (e.g. pasture land, fallen trees) along its straight line. Because we expected linearity to increase under conditions of increased knowledge, we calculate the accumulated number of visits to a given goal (i.e. end location of a travel bout) prior to the current travel bout. Similarly, we expected linearity to increase under conditions of increased intragroup competition and hunger levels. Thus, we estimated intragroup competition as the number of individuals present in the group at the moment of travelling and hunger as the inverse of the accumulated number of minutes that the group had been feeding that day prior to the travel bout (i.e. inverse to satiation state; Plante et al., 2014; Jang et al., 2019; Salmi et al., 2020).

We determined the presence of nodes by examining the locations where two or more habitual route segments intercepted each other. If such interception was followed by at least one change in direction larger than 45 deg within the next 15 m, we consider the intersection as a node (Presotto et al., 2018) (Fig. S4). Subsequently, we generated correlation matrices describing the connections between all pairs of nodes within the same network weighted by the number of times that the connection was used to travel and the distance (m) between nodes. We used the R package igraph 1.2.4.2 (Csardi and Nepusz, 2006) to calculate metrics derived from the structure of each network (Barthélemy, 2011). First, we estimated the strength of individual nodes within each network using the function strength, which calculates the mean number of travel events that occurred across all interconnected pairs of nodes within the network (Barthélemy, 2011; Soh et al., 2010). Second, we calculated the shortest path length to travel from each node to all other nodes within the network using the function shortest.paths on the matrix of connections weighted by the frequency of travel between each pair of connections (Barthélemy, 2011). Because the study groups navigated route networks of different sizes, we weighted the mean shortest path lengths by the diameter of the network (i.e. the shortest distance between the two most distant nodes in the network), which was calculated using the diameter function. Third, we calculated the centrality of each network as its closeness (i.e. average distance of each node to all others) weighted by the frequency of travel between each pair of nodes using the function closeness (Borgatti, 2005).

### Statistical analysis

To test each of the hypotheses associated with the use of cognitive maps, we ran a series of linear mixed models (LMMs). We fitted all models in R (version 4.0.1, https://www.r-project.org/) using the function lmer from the package lme4 (version 1.1-23; Bates et al., 2015) except for the linearity model (explained below). For all models, we controlled for the influence of repeated measures including group ID as random effect. We used a binary predictor variable differentiating between observed (0) and simulated (1) ranging patterns. We included random slopes for the predictor variables to keep type I error rates at a nominal level of 5% (Barr et al., 2013).

First, we fitted two LMMs to test for differences between simulated agents and black howlers in their tendency to overlap travel paths. As response variables, we selected either: (1) the ratio of weekly distance travelled within the habitual route network (overlap model 1), or (2) the frequency of use for each habitual route segments (overlap model 2). Second, we ran two LMMs to test whether simulated and observed movement patterns differed in the number of directions used to arrive to and depart from revisited locations. We selected as response variables: (1) the number of different directions to reach and depart from revisited feeding trees (angles model 1), and (2) the frequency with which each direction was used. Third, we ran three LMMs to test for differences in the structure of observed and simulated route networks, selecting as response variables: (1) the strength of the network at node level, (2) the mean shortest path length weighted by the diameter of the network, and (3) the degree of centrality of the network at node level.

Additionally, we ran a Kruskal–Wallis test to examine differences in the linearity of the travel trajectories among the study groups. In addition, we designed a GLMM with beta error structure and logit link function to examine the ability of black howler monkeys to increase the linearity of their travel bouts using the function glmmTMB from the R package glmmTMB (Brooks et al., 2017; based on Jang et al., 2019). In this model, the predictor variables included the accumulated number of prior visits (i.e. proxy for knowledge), accumulated minutes feeding prior departure (i.e. proxy for hunger), group size at the moment of travelling (i.e. proxy for intragroup competition) and activity upon arrival (i.e. resting, foraging, howling). We tested for a two-way interaction between group size and hunger because we hypothesized that black howlers would travel increasingly linearly with increasing intragroup competition, but only when their hunger levels were higher. We included control predictors for straight-line distance and the proportion of the travel bout that fell within the route network. We determined the significance of individual effects by dropping them from the model one at a time.

For all model estimates, we determined 95% confidence intervals by bootstrapping (1000 replicates), using the functions bootMer of the lme4 package and simulate.glmmTMB of the glmmTMB package, respectively. We checked for model stability by excluding groups one at a time from the dataset and fitting the same models to these subsets, which showed no indication of the presence of influential groups.

All means are presented ±s.e.m. unless otherwise indicated.

## RESULTS

**Fig. 2.**
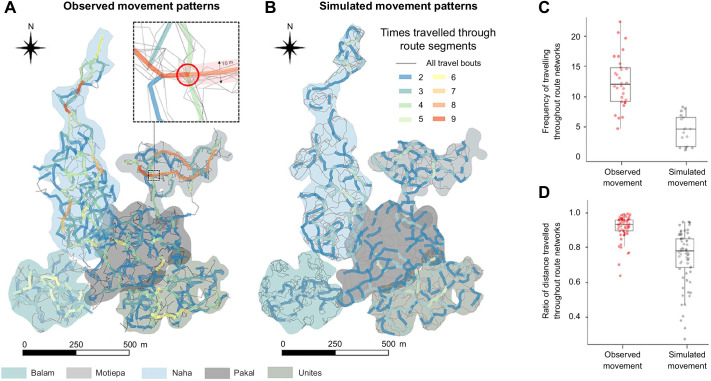
**Comparing the tendency to travel along paths used only once between five groups of black howler monkeys at Palenque National Park, Mexico, and five simulated groups.** (A,B) Route networks based on (A) observed and (B) simulated (correlated random walk) ranging patterns. (C,D) Boxplot graphs indicate (C) the frequency of use of each habitual route segment and (D) the weekly ratio of distance travelled within route networks for observed and simulated ranging patterns.

The mean length of an individual travel bout was 65.3±57.5 m, while the mean daily path length was 365.8±199.2 m (range: 28.3–1022.8 m). Black howler groups overlapped between 84% and 92% of the total distance travelled (mean observed route network length: 3.5±1.1 km; Fig. 2A), whereas simulated random-walk agents overlapped between 58% and 65% of the total distance travelled (mean simulated route network length: 2.7±0.6 km; Fig. 2B). Both black howlers and simulated agents travelled a larger proportion of their weekly paths inside (black howlers: mean=0.89±0.07; simulated agents: mean=0.71±0.17) than outside (black howlers: mean=0.09±0.05; simulated agents: mean=0.28±0.15) habitual route segments. However, black howlers showed a significantly higher proportion of distance travelled inside their habitual route networks than did simulated random-walk agents (LMM: likelihood ratio test: χ^2^=8.281, d.f.=1, *P*=0.004; Fig. 2C). Similarly, while black howler monkeys reused habitual route between 2 and 9 weeks, simulated agents reused habitual route segments in between 2 and 5 weekly sampling units. Indeed, we found that the frequency of use of habitual route segments was higher for black howlers (mean=12.3±3.9 day^−1^) than for simulated agents (mean=4.2±2.3 day^−1^; likelihood ratio test: χ^2^=12.458, d.f.=1, *P*<0.001; Fig. 2D).

**Fig. 3.**
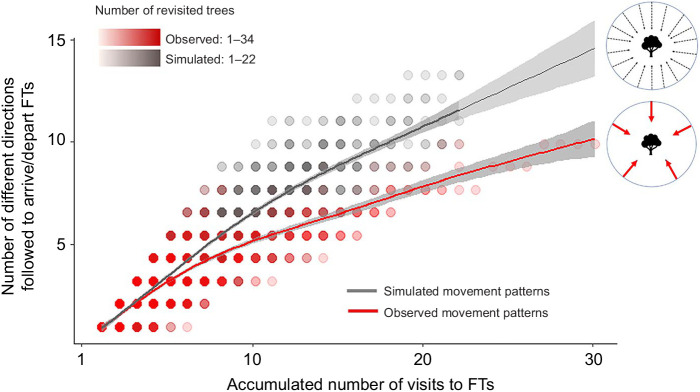
**Number of directions used to reach and depart from revisited feeding trees (FTs) for simulated and observed ranging patterns of five black howler monkey groups at Palenque National Park, Mexico.** The lines represent the fitted model (angles model 1), shaded areas represent the 95% confidence intervals of the fitted model and shaded dots represent the sample size for each number of visits.

Black howlers showed a tendency to approach and depart from revisited feeding trees through a lower number of directions (mean=4.53±1.44; *N*=156 revisited feeding trees) in comparison with simulated agents (mean=7.59±1.96; likelihood ratio test: χ^2^=11.416, d.f.=1, *P*<0.001; Fig. 3). In addition, black howlers used each travel direction to reach revisited feeding trees more frequently (mean=2.02±0.63) than simulated movement agents (mean=1.67±0.31; likelihood ratio test: χ^2^=5.639, d.f.=1, *P*=0.018).

Linearity values did not differ among study groups (Kruskal–Wallis test: *H=*5.32, d.f.=4, *P=*0.26) and remained relatively constant throughout the study period (mean monthly range: 0.64–1.0; *N*=657 travel bouts). The comparison between the full and the null models showed that black howlers did not optimize the linearity of their trajectories as a function of either the accumulated number of visits, increasing hunger levels within the day, or changes in group size (likelihood ratio test: χ^2^=6.664, d.f.=8, *P*=0.573; Table S1).

**Fig. 4.**
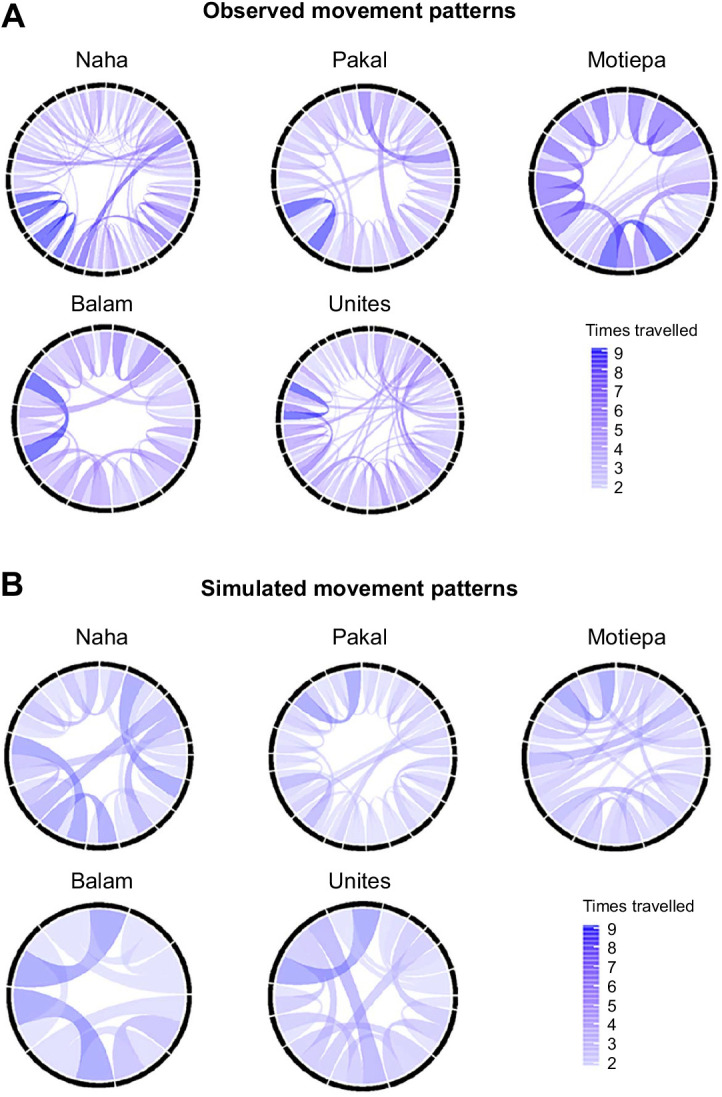
**Each habitual route network for both observed and simulated movement patterns represented as polar graphs.** (A) Observed movement patterns; (B) simulated movement patterns. Names above polar plots indicate study group. Each string within a circle represents the connection between a pair of nodes from the network. The colour gradient represents the number of times the study group or agent travelled along each connection and the string width is proportional to the importance of the connection between pairs of nodes within the network (i.e. number of times travelled between nodes divided by the total number of nodes in the network).

We detected a total of 132 nodes across the five observed route networks (mean=25.6±8.1 nodes per group) and 85 nodes across the five simulated route networks (mean=14.4±5.5 nodes per group). Nodes located within observed and simulated networks were interconnected by a mean of 2.8±0.2 and 2.4±0.3 habitual route segments, respectively (Fig. 4). Our GLMMs revealed significant differences between observed and simulated route networks at structural levels where observed route networks showed higher levels of strength (mean=9.51±4.37) than simulated networks (mean=6.24±2.67; likelihood ratio test: χ^2^=7.378, d.f.=1, *P*=0.006). The mean shortest path in relation to network diameter was longer in simulated route networks (mean=0.49±0.28) than observed route networks (mean=0.37±0.21; likelihood ratio test: χ^2^=5.363, d.f.=1, *P*=0.021). Finally, observed route networks showed a lower degree of closeness (mean=1.43×10^−3^±0.79×10^−3^) than simulated route networks (mean=8.86×10^−3^±7.92×10^−3^; likelihood ratio test: χ^2^=8.168, d.f.=1, *P*=0.004).

## DISCUSSION

In this study, we used a novel approach to investigate navigation strategies of black howler monkeys by comparing observed movement patterns with computer-generated random movement patterns, which acted as a control. Movement patterns by black howlers showed remarkable differences from simulated movement patterns, as black howlers frequently travelled along habitually used route segments and reached revisited feeding trees from a limited number of directions. In addition, black howler monkeys did not travel increasingly more linearly with increasing experience travelling towards these specific goals, intragroup competition (group size) or internal motivation (hunger), suggesting constrained cognitive abilities. While these findings discard the use of a Euclidean cognitive map in black howler monkeys, the highly efficient structure of the observed networks in comparison to simulated networks suggests that black howler monkeys rely on a labelled graph cognitive map rather than a topological map to navigate.

Even though we found that, through both observed and simulated movement patterns, black howler monkeys tended to navigate more frequently within than outside their respective network of habitual routes, we found remarkable differences in the use of habitual routes. Not only was the ratio of distance travelled within the route network higher in observed movement patterns than in simulated ones, but so was the frequency with which habitual route segments were used. Black howler monkeys showed a clear asymmetry in the frequency of use of route segments, travelling through the same sequence of trees between 2 and 9 weeks. Contrary to the observed data, simulated agents never travelled through the same segment on more than four different occasions. The high frequency of use of habitual route segments by black howler monkeys in specific locations might reflect a response to local advantages such as reduced energetic expenditure or availability of food resources (Presotto and Izar, 2010; Presotto et al., 2018; de Guinea et al., 2019; Green et al., 2020). Even though simulated paths did not overlap as much as observed paths, there was enough inter-path overlap to generate a route network that could be compared with the observed route networks. Such a relatively high degree of overlap among the simulated paths can be interpreted as a reliable representation of black howlers' movement patterns that successfully mimicked real movement patterns (Garber and Hannon, 1993). It is likely that incorporating the resistance of the landscape in the simulations (e.g. terrain's slopes) could increase even more the overlap among simulated travel paths. Previous research at our field site revealed that black howler monkeys locate habitual route segments in areas that minimize the energetic cost of travelling while favouring the visual monitoring of potential food resources (de Guinea et al., 2019; see also Hopkins, 2011, 2013). Incorporating landscape attributes and the location of food resources in the movement decisions of the simulated agent is the next step to infer the level of cognition involved in the cognitive maps of black howler monkeys. Similarly, simulating random movement patterns within the observed route network of the study groups has the potential of elucidating black howler monkeys' spatiotemporal knowledge of food resources (Suarez et al., 2014). Animals navigating within a habitual route network are expected to encounter nutritionally valuable food resources at higher rates than simulated random walk agents would do, even within the same route network. If that is the case, this would be direct evidence of navigation efficiency and spatial cognition in the observed animals.

In line with the high tendency of overlap among travel paths, black howler monkeys moved towards and away from revisited feeding trees from the same set of directions and, therefore, tended to navigate using a limited number of routes. In contrast, simulated agents kept accumulating different arrival and departure directions over time (Garber and Hannon, 1993). Arboreality may impose spatial constraints triggered by the forest structure, such as inter-tree connectivity (Hopkins, 2011; McLean et al., 2016). For instance, the proximity of pasturelands, physical obstacles (i.e. water bodies, Mayan ruins) or canopy gaps could occasionally be constraining the accessibility options to specific revisited feeding trees (de Guinea et al., 2019; Fig. S5). Even though we cannot discard the effect of constrained accessibility to specific trees, our results indicate that all study groups consistently reached a plateau in the number of different directions used to approach revisited feeding trees. In addition, we explicitly demonstrated that black howler monkeys were unable to optimize the linearity of their trajectories under strict conditions of continuous forest coverage. Despite the benefits associated with optimizing the trajectory of travel bouts such as gaining rapid access to fruits, especially under conditions of increasing hunger or competition (Jang et al., 2019), black howler monkeys consistently deviated from a straight-line path when travelling. Hence, the travel patterns of black howler monkeys were consistently associated with route-based cognitive maps, which likely dictated the shape of their travel trajectories mirroring the shape of the route network that they were travelling through.

Establishing a set of habitual routes to navigate can be advantageous in predictable environments where seasonal variability of food resources is not especially marked (Fagan et al., 2013; Riotte-Lambert and Matthiopoulos, 2020). In such scenarios, memorizing the location of a habitual route network might be more advantageous than constantly memorizing and forgetting the location of rare and ephemeral food resources (Tello-Ramos et al., 2019). Thus, both Euclidean cognitive maps and route-based cognitive maps can provide benefits depending on the spatiotemporal dynamics of the landscape in which the species lives. In addition, by consistently travelling through habitual routes, the cost of travelling might decrease because the route itself becomes clearer and smoother for the traveller (Shepard et al., 2013). Even animal species with known sophisticated spatial skills, such as humans (*Homo sapiens*) and chimpanzees (*Pan troglodytes*), navigate through urban areas (Byrne, 1979), rainforests (Jang et al., 2019; Green et al., 2020) or even virtual reality scenarios (Foo et al., 2005) using habitual routes or trails to minimize the cost of travelling. Alternatively, animals navigating through habitual routes may rely on visual memories associated with salient features of the landscape such as landmarks (i.e. cliffs, emergent trees, ridges; Noser and Byrne, 2015; Presotto et al., 2018), suggesting that the cognitive process involved might be associative learning rather than path optimisation (Fernandes et al., 2018). Even though we did not explicitly test for the effect of visual memories in the movement decisions of black howler monkeys, previous research has shown an association between habitual routes and energy-saving terrain at Palenque National Park (de Guinea et al., 2019). Thus, while black howler monkeys likely benefit from optimizing the location of habitual routes over the course of multiple generations to minimize the cost of travelling across such a variant landscape, there may still be an effect of visual memories in the movement decisions of black howler monkeys that remains unexplored.

Lastly, we aimed to explore whether black howler monkeys incorporate metric information in the construction of their route network to determine the use of either a topological or a labelled graph cognitive map. We found that observed and simulated route networks differed in all the parameters examined as indicators for network structure and efficiency. The involvement of each individual node in the overall activity of the network (i.e. strength) was higher in the observed compared to the simulated route networks. It is likely that black howler monkeys travelled more frequently between a higher number of combination of pairs of nodes than did simulated agents (Barthélemy, 2011). Such high values of strength indicate that while simulated agents travelled homogeneously across their network, black howlers were highly selective in the nodes they used to travel. Similarly, we found that the mean shortest path between all pairs of nodes relative to the network's diameter was shorter for black howler monkey route networks than for simulated networks. This is the most direct evidence of the efficiency of the observed route networks in which nodes and connections are strategically located and combined to favour rapid travel throughout the entire home range (Soh et al., 2010). Likely, black howler monkeys incorporated knowledge on distance and angles among biologically meaningful locations within their home range when developing their route network to promote efficient travelling among distant areas. In addition, the low degrees of centrality in real route networks suggested that black howlers did not depend on few, specific trees to bridge different areas within their home range. Instead, they connected distant locations through multiple different routes, probably owing to the structural dynamics of rainforests characterized by frequent tree falls (Martinez-Ramos et al., 1998). Non-centralized route networks in arboreal tropical species would be highly advantageous to avoid losing access to specific areas and to facilitate the process of finding alternative routes once a route becomes impassable (Jacoby and Freeman, 2016).

In conclusion, our results strongly support the hypothesis that black howlers incorporate metric information about the environment when constructing their route networks to enhance their navigation efficiency. Potentially, with time, black howler groups might accumulate information regarding the shape and location of habitual routes, which could be optimized through experience (e.g. exploration, innovative detours) and transferred intergenerationally through social learning (Dean et al., 2014; Sasaki and Biro, 2017). Yet, more direct evidence on the movement decisions of black howler monkeys is needed to reach a robust conclusion on the use of a labelled graph map and the potential optimization of their route networks through time. We propose future research to extend the collection of ranging and behavioural data over several generations to be able to determine changes in the structure of the observed route networks across generations. Incorporating additional layers of geographic information (i.e. elevation, forest structure, terrain slopes) and spatiotemporal variability of food resources and applying these to both long-term observed movement and correlated-random walk simulations will provide a realistic scenario to examine the role of cognitive processes during path optimization. Alternatively, we suggest examining the structural efficiency of networks by estimating the energetic costs of travelling along each combination of nodes using layers of geographic information (Halsey, 2016). By comparing the energetic cost of the actual travel choice of a study group against the energetic costs of all possible options to travel between these locations, we would obtain direct evidence of the animal's ability to select the most beneficial path by incorporating metric information into its movement decisions (Gallotti et al., 2016; Teichroeb and Smeltzer, 2018).

## Supplementary Material

Supplementary information
