# Supplementary material for: Cognitive maps in the wild: revealing the use of metric information in black howler monkey route navigation
Source: J Exp Biol. 2021 Aug 13;224(15):jeb242430. doi: 10.1242/jeb.242430 (PMC8380465; doi:10.1242/jeb.242430)
Supplement: Supplementary information [file jexbio-224-242430-s1.pdf]

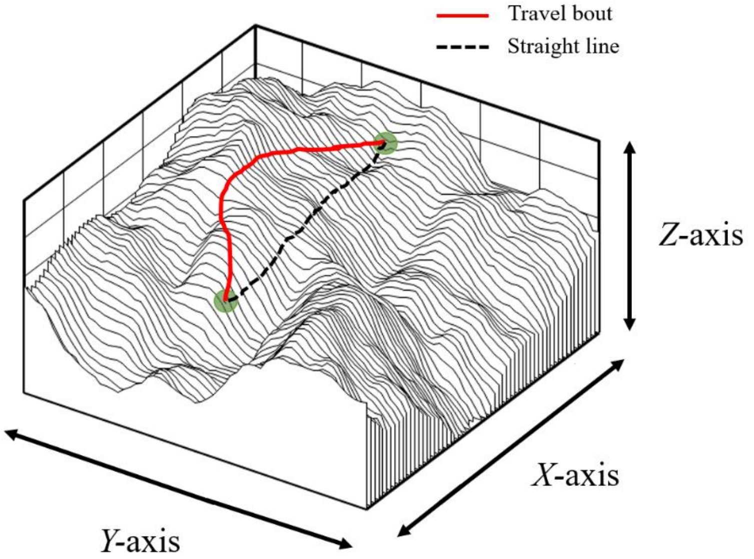

$$linearity = \frac{\sum_{t=0}^{n} \sqrt{(x_{t_0} - x_{t_0+1})^2 + (y_{t_0} - y_{t_0+1})^2 + (z_{t_0} - z_{t_0+1})^2}}{\sqrt{(x_{t_0} - x_{t_n})^2 + (y_{t_0} - y_{t_n})^2 + (z_{t_0} - z_{t_n})^2}}$$

Fig. S1. Linearity calculated as the coefficient between the actual distance travelled (red line) and the straightest line between two points (black dotted line). In the equation, the starting location is represented as x_{t0}, y_{t0} and z_{t0}, and each step is represented as x_{t0+1}, y_{t0+1} and z_{t0+1}.

Journal of Experimental Biology • Supplementary information

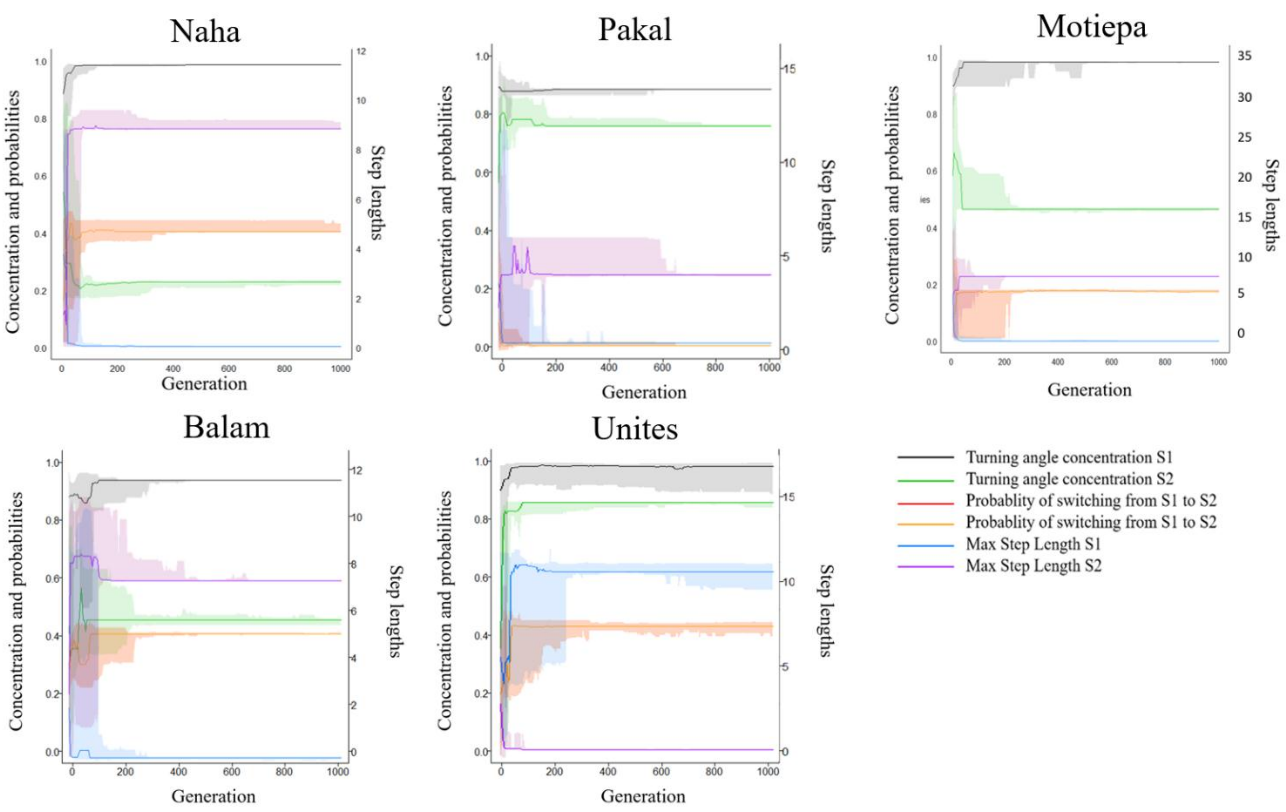

Fig. S2. Evolution of the optimised set of input parameters along the “adjustModel” (“SiMRiv” 1.0.3 R-package) algorithm’s generation. These plots determine that the final solutions converged into stable values indicating that the algorithm succeeded in replicating the real trajectory.

Journal of Experimental Biology • Supplementary information

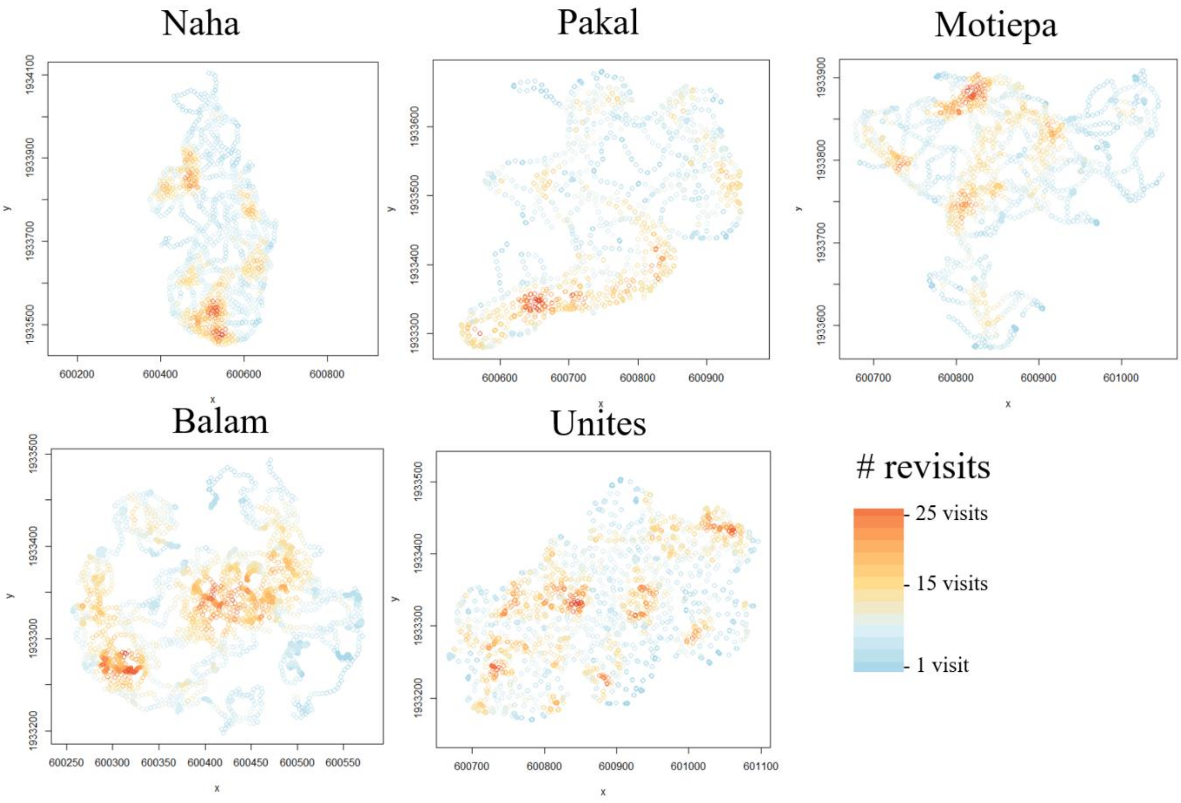

Fig. S3. Number of times that each simulated correlated-random walk agent visited the same location within the constraints of the home range of the study group. Calculations were done using the R-package *recurse* and with the function *getRecursions* (Bracis et al., 2018).

Journal of Experimental Biology • Supplementary information

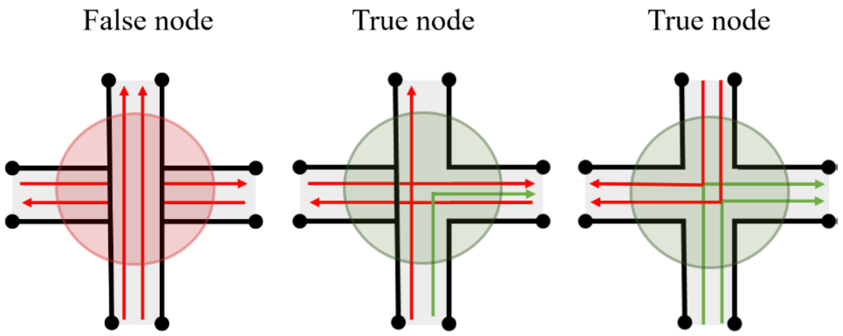

Fig. S4. Criteria selected to determine whether the intersection between habitual route segments represents a node.

Journal of Experimental Biology • Supplementary information

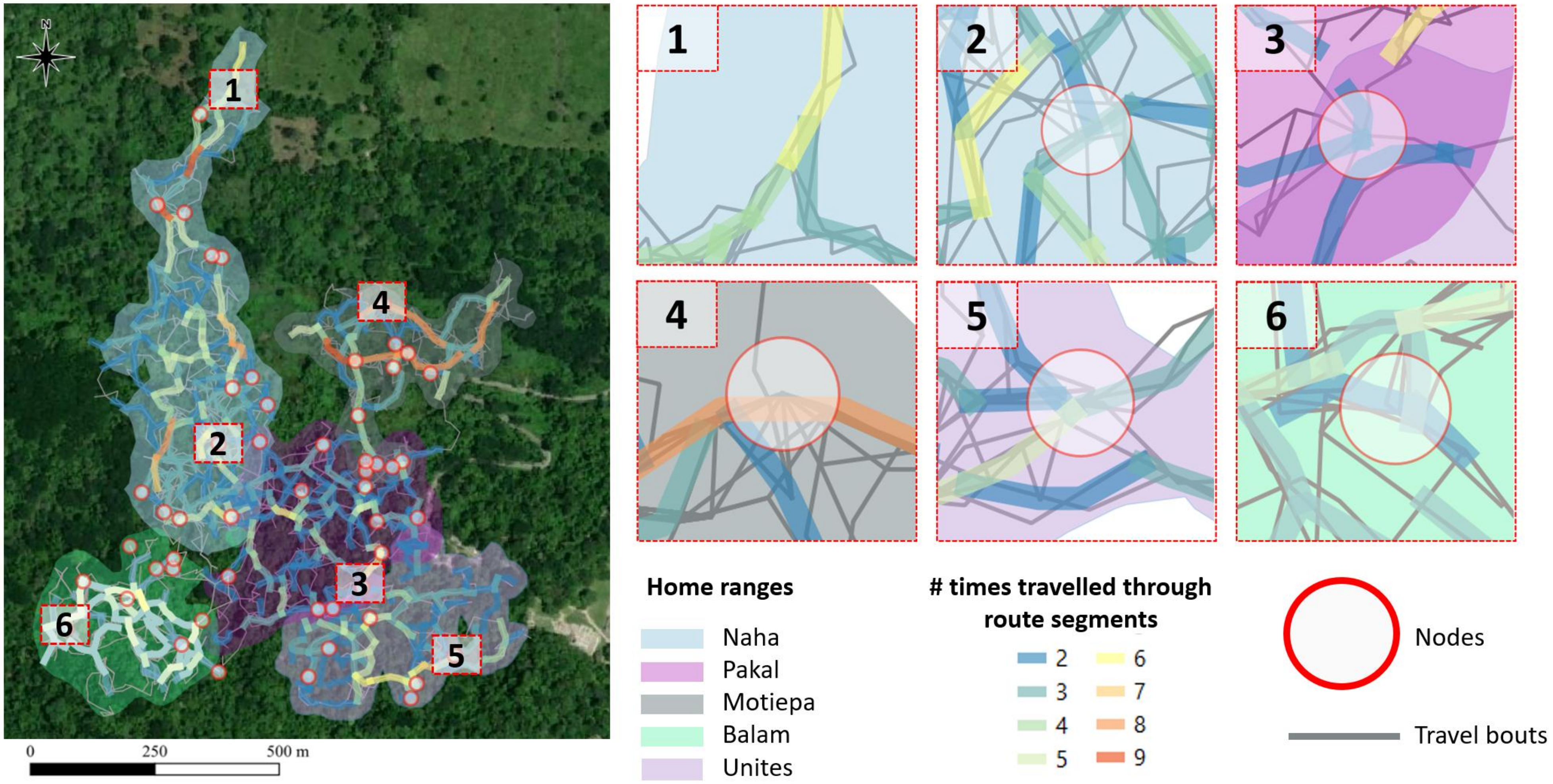

Fig. S5. Recorded travel paths (grey) describing habitual routes and nodes used by black howler monkeys in Palenque National Park overlaid over a satellite image taken by Sentinele 2 in August 2017 (30m^2 resolution). On the right, there is a panel of six examples illustrating that individual travel bouts overlap in habitual route segments and intersections determine the location of nodes.

Table S1. Results of the GLMM testing whether black howlers at PNP changed their travel linearity under variant conditions of knowledge (i.e., accumulated number of visits), hunger, intragroup competition (i.e., group size), behavioural upon arrival and their interactions.

Predictor variable	Estimate	SE	CI_{lower}	CI_{upper}	*p*-value
(Intercept)	1.444	0.233	0.118	0.413	< 0.001
Accumulated number of visits	-0.007	0.041	-0.089	0.074	0.859
Hunger	0.053	0.039	-0.025	0.0131	0.181
Group size	-0.047	0.046	-0.137	0.044	0.311
Behaviour upon arrival	-0.237	0.272	-0.713	0.311	0.643
Group size * Hunger	0.039	0.038	-0.036	0.114	0.312
Proportion within route network [a]	-0.099	0.039	-0.176	-0.021	0.012
Straight line distance [a]	-0.095	0.040	-0.174	-0.017	0.016

[a] Represent control predictors included in the model

Journal of Experimental Biology • Supplementary information
